# Supplementary material for: Fetal Oxygenation from the 23rd to the 36th Week of Gestation Evaluated through the Umbilical Cord Blood Gas Analysis
Source: Int J Mol Sci. 2023 Aug 6;24(15):12487. doi: 10.3390/ijms241512487 (PMC10419490; doi:10.3390/ijms241512487)
Supplement: Supplementary file 1 [file ijms-24-12487-s001.zip › supplementary captions.pdf]

Supplementary Figure S1. Umbilical cord oxygenation status (venous pO_2, panel A; arterial pO_2, panel B; fetal oxygen extraction, panel C) stratified by groups of gestational age. * = $p < 0.05$; ** = $p < 0.01$; *** = $p < 0.001$. Data are expressed as median and IQR.

Supplementary Figure S2. Umbilical cord hemoglobin levels (venous hemoglobin, panel A; arterial hemoglobin, panel B) and fetal venous oxygen content (panel C) stratified by groups of gestational age. * = $p < 0.05$; ** = $p < 0.01$; *** = $p < 0.001$. Data are expressed as median and IQR.

Supplementary Figure S3. Umbilical cord pH (venous pH, panel A; arterial pH, panel B), BE levels (venous BE, panel C; arterial BE, panel D), and bicarbonate (venous bicarbonate, panel E; arterial bicarbonate, panel F) stratified by groups of gestational age. * = $p < 0.05$; ** = $p < 0.01$; *** = $p < 0.001$. Data are expressed as median and IQR.

Supplementary Figure S4. Umbilical cord carbon dioxide levels (venous pCO_2, panel A; arterial pCO_2, panel B; fetal CO_2 production, panel C) stratified by groups of gestational age. * = $p <0.05$; ** = $p < 0.01$; *** = $p < 0.001$. Data are expressed as median and IQR.

Supplementary Figure S5. Umbilical cord lactate levels (venous lactate, panel A; arterial lactate, panel B; fetal lactate production, panel C) stratified by groups of gestational age. * = $p < 0.05$; ** = $p < 0.01$; *** = $p < 0.001$. Data are expressed as median and IQR.
