# Supplementary figures and images for: Fetal Oxygenation from the 23rd to the 36th Week of Gestation Evaluated through the Umbilical Cord Blood Gas Analysis
Source: Int J Mol Sci. 2023 Aug 6;24(15):12487. doi: 10.3390/ijms241512487 (PMC10419490; doi:10.3390/ijms241512487)

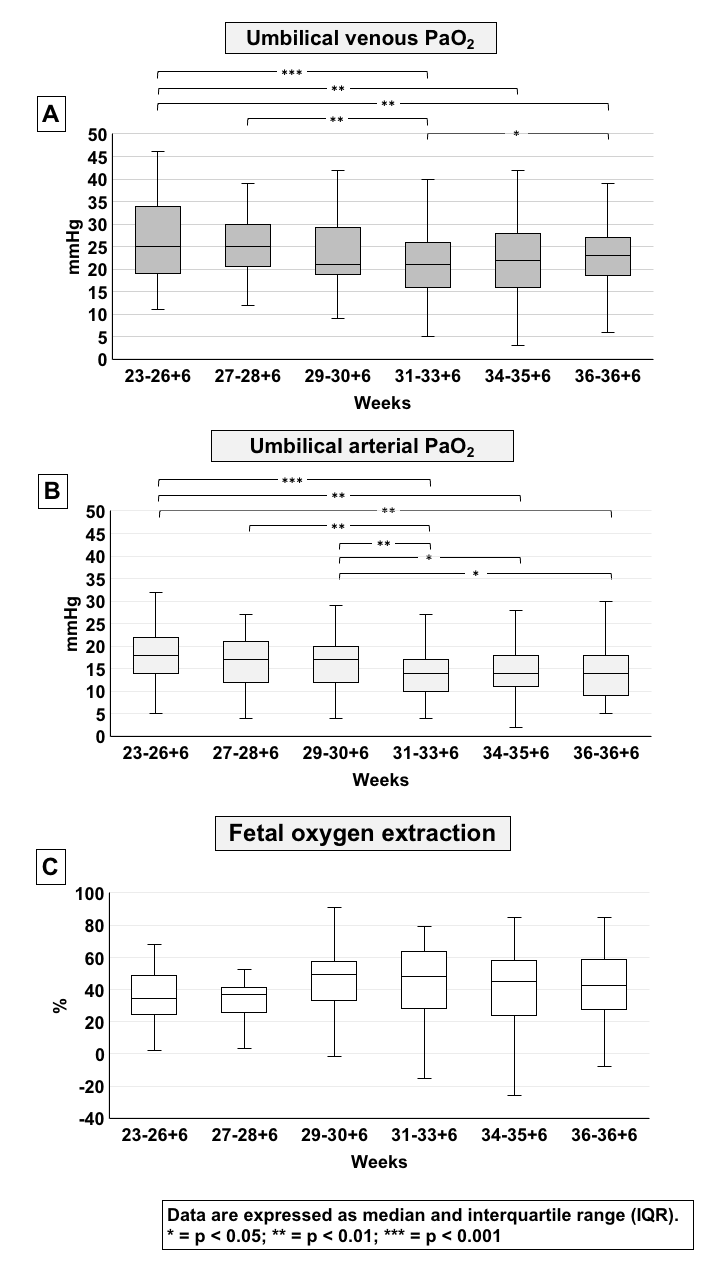

Supplement: Supplementary file 1 [file ijms-24-12487-s001.zip › REVISED Supplementary Figure S1.tiff]

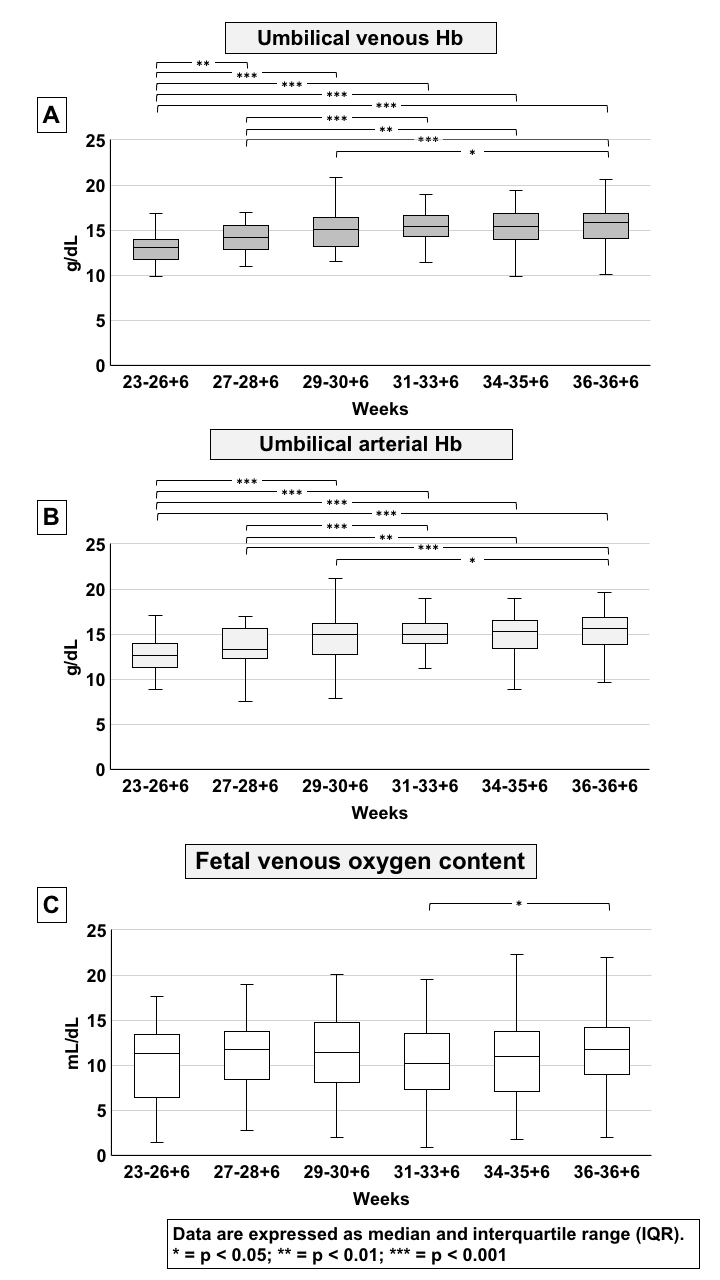

Supplement: Supplementary file 1 [file ijms-24-12487-s001.zip › REVISED Supplementary Figure S2.tiff]

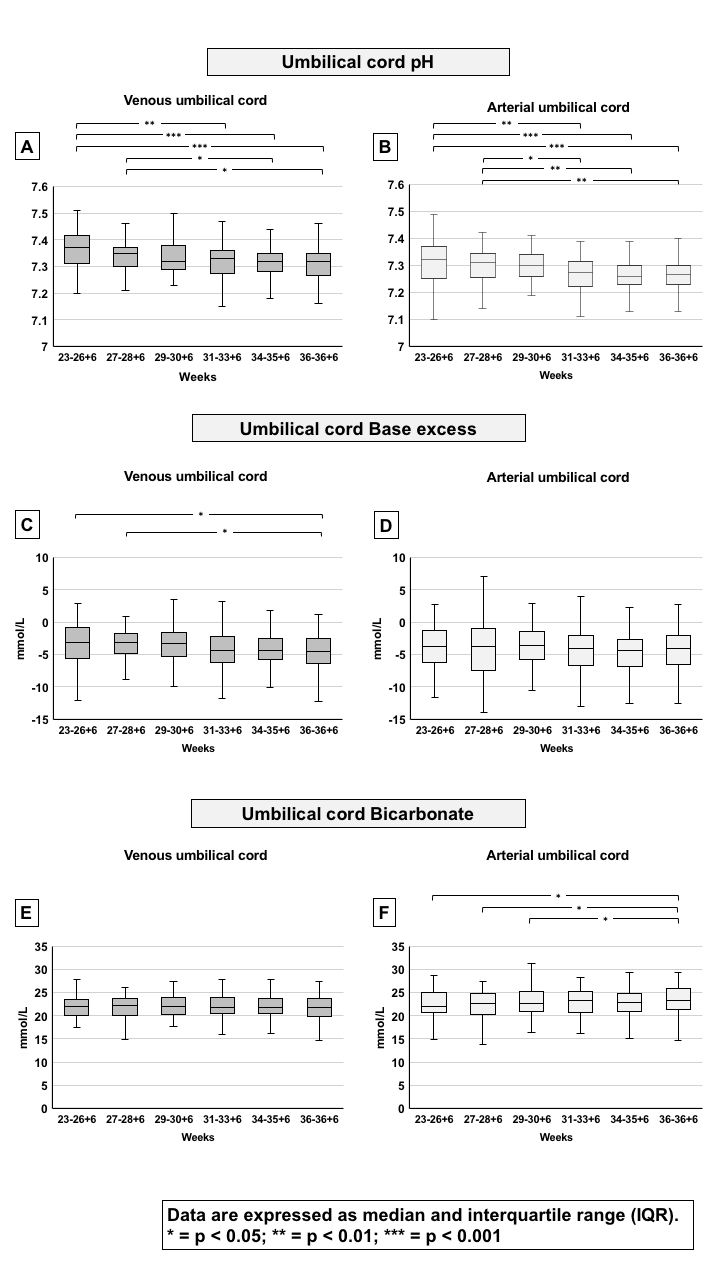

Supplement: Supplementary file 1 [file ijms-24-12487-s001.zip › REVISED Supplementary Figure S3.tiff]

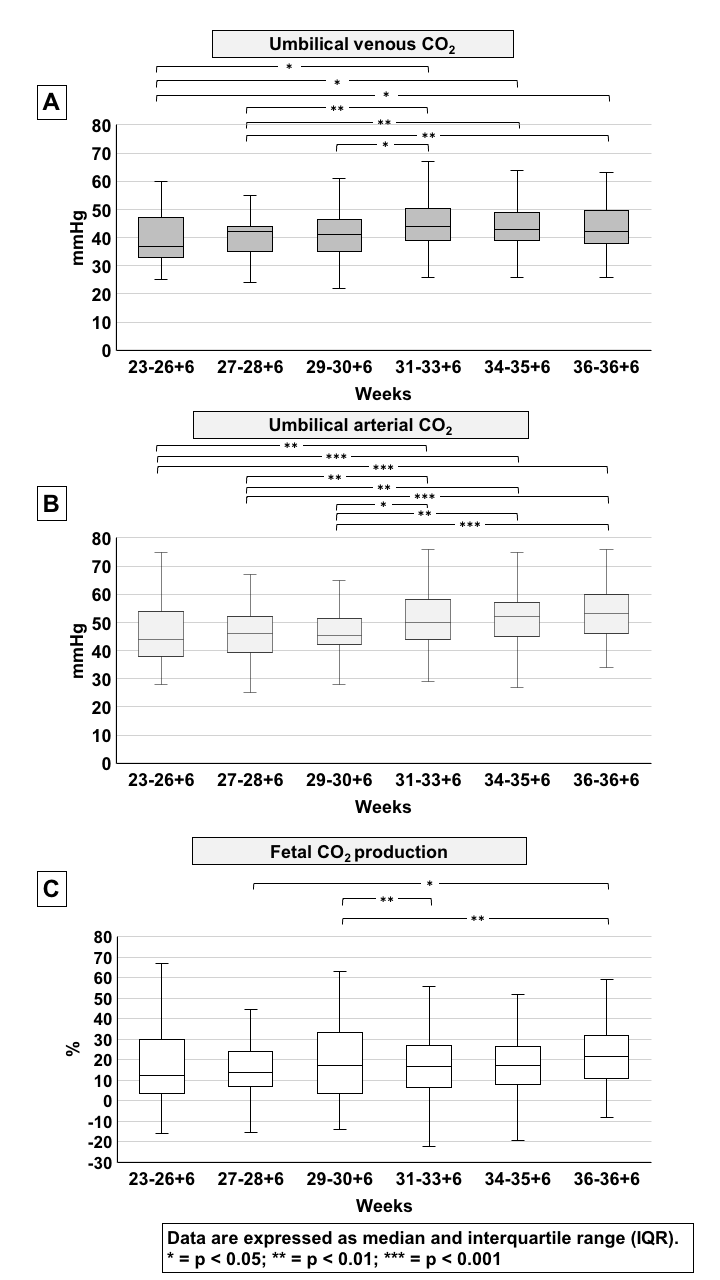

Supplement: Supplementary file 1 [file ijms-24-12487-s001.zip › REVISED Supplementary Figure S4.tiff]

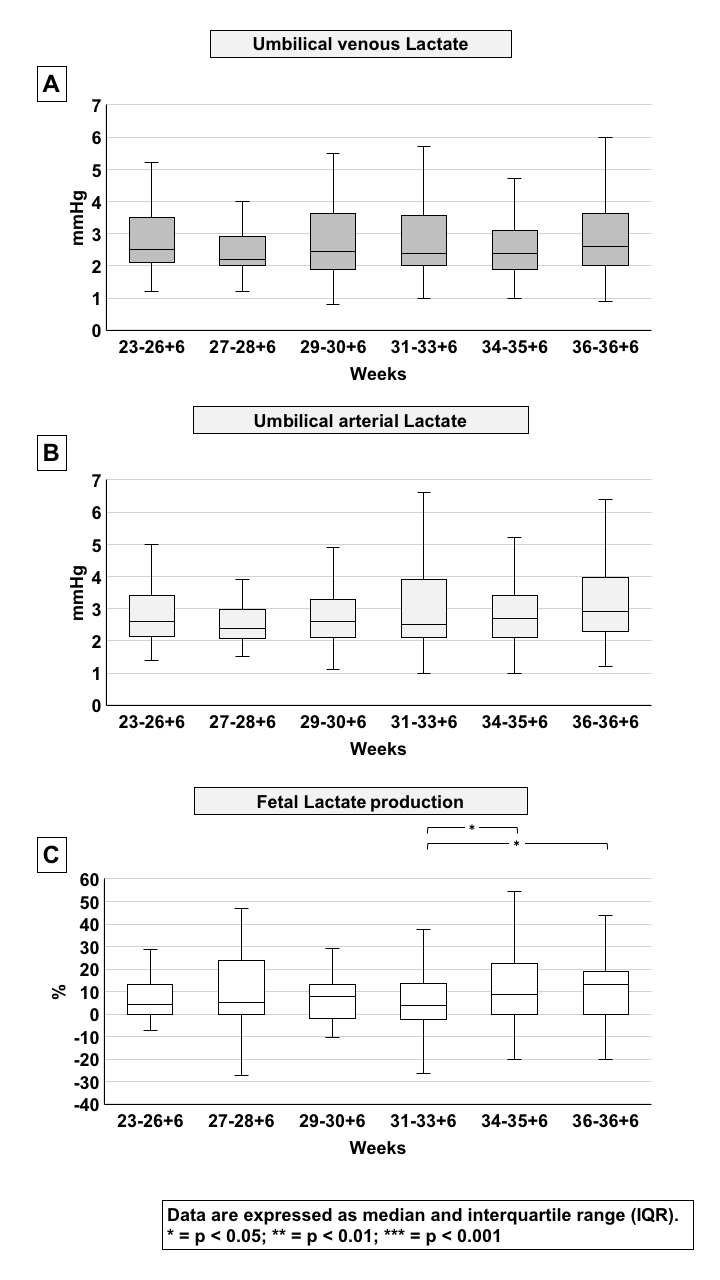

Supplement: Supplementary file 1 [file ijms-24-12487-s001.zip › REVISED Supplementary Figure S5.tiff]
